# Structural Magnetic Resonance Imaging Correlates of Aggression in Psychosis: A Systematic Review and Effect Size Analysis

**DOI:** 10.3389/fpsyt.2018.00217

**Published:** 2018-06-07

**Authors:** Sonja Widmayer, Julia F. Sowislo, Hermann A. Jungfer, Stefan Borgwardt, Undine E. Lang, Rolf D. Stieglitz, Christian G. Huber

**Affiliations:** ^1^Erwachsenen-Psychiatrische Klinik, Universitäre Psychiatrische Kliniken Basel, Basel, Switzerland; ^2^Klinik für Psychiatrie und Psychotherapie, Universitätsklinikum Hamburg-Eppendorf, Hamburg, Germany; ^3^Asklepios Klinik Nord-Ochsenzoll, Hamburg, Germany; ^4^Fakultät für Psychologie, Universität Basel, Basel, Switzerland

**Keywords:** aggression, psychosis, structural magnetic resonance imaging, systematic review, effect size analysis

## Abstract

**Background:** Aggression in psychoses is of high clinical importance, and volumetric MRI techniques have been used to explore its structural brain correlates.

**Methods:** We conducted a systematic review searching EMBASE, ScienceDirect, and PsycINFO through September 2017 using thesauri representing aggression, psychosis, and brain imaging. We calculated effect sizes for each study and mean Hedge's g for whole brain (WB) volume. Methodological quality was established using the PRISMA checklist (PROSPERO: CRD42014014461).

**Results:** Our sample consisted of 12 studies with 470 patients and 155 healthy controls (HC). After subtracting subjects due to cohort overlaps, 314 patients and 96 HC remained. Qualitative analyses showed lower volumes of WB, prefrontal regions, temporal lobe, hippocampus, thalamus and cerebellum, and higher volumes of lateral ventricles, amygdala, and putamen in violent vs. non-violent people with schizophrenia. In quantitative analyses, violent persons with schizophrenia exhibited a significantly lower WB volume than HC (*p* = 0.004), and also lower than non-violent persons with schizophrenia (*p* = 0.007).

**Conclusions:** We reviewed evidence for differences in brain volume correlates of aggression in persons with schizophrenia. Our results point toward a reduced whole brain volume in violent as opposed to non-violent persons with schizophrenia. However, considerable sample overlap in the literature, lack of reporting of potential confounding variables, and missing research on affective psychoses limit our explanatory power. To permit stronger conclusions, further studies evaluating structural correlates of aggression in psychotic disorders are needed.

## Introduction

### Aggression in persons with psychotic disorders

#### Defining aggression

Aggression, defined as hostile or destructive behavior, can be classified by the target of aggression (self-directed or directed at others), the mode of aggression (physical or verbal), or the cause of aggression (1).

We distinguish premeditated vs. impulsive aggression. Premeditated aggression represents a planned behavior while impulsive aggression occurs as a response to provocation or stress (2, 3). Impulsive aggression following a dangerous threat is part of normal defensive behavior—if the aggressive response is exaggerated in relation to the provocation, impulsive aggression becomes pathological (1).

#### Epidemiology and risk factors of aggression in persons with psychotic disorders

Persons with psychoses are at increased risks for violent behavior (4–6) even in the first episode of illness (7–10) and in at-risk mental states (11), although more than 90% of violent acts in society are committed by persons without psychoses (12). Brekke et al. (13) even found that people with schizophrenia are at a greater risk of becoming victims of violence than of being an offender. Still, according to Wehring and Carpenter (14), a focus on criminal records underestimates the prevalence of aggressive behavior in schizophrenia.

In a meta-analysis including 110 studies, Witt et al. (15) examined risk factors of violence in persons with psychotic disorders. About 88% of the persons in the included studies had been diagnosed with schizophrenia. The authors found the following elements to be associated with violence risk: hostile behavior, poor impulse control, lack of insight, recent alcohol or drug misuse, and non-adherence with psychological or pharmacological therapies (15). Criminal history was more strongly associated with violence than substance misuse or demographic factors (15). Moreover, economic deprivation, violent victimization, childhood conduct problems, and sexual abuse are known common risk factors favoring aggression (16). Witt et al. (15) reported victimization to be one of the strongest risk factors.

### Structural MRI findings in psychoses

#### Structural brain alterations in persons with psychoses

Schizophrenia is associated with ventricular enlargement and reductions in frontal and temporal lobe gray matter (GM) (17, 18). Furthermore, structural abnormalities in schizophrenia appear to include a smaller amygdala, hippocampus, and parahippocampus (19, 20). Goodkind et al. (21) performed a large-scale voxel-based meta-analysis on GM abnormalities in different psychiatric diseases including schizophrenia. They reported increased GM in the striatum in patients as opposed to controls. Decreased GM was observed in the bilateral anterior insula, dorsal anterior cingulate (AC), dorsomedial prefrontal cortex, ventromedial prefrontal cortex, thalamus, amygdala, hippocampus, superior temporal gyrus, and parietal operculum.

A meta-analysis indicated the occurrence of GM reductions in temporal, AC, cerebellar, and insular regions, in a first psychotic episode (22).

Strakowski et al. (23) detected increased volumes of amygdala, thalamus, and globus pallidus in affective psychoses. Altshuler et al. (24) and Brambilla et al. (25) confirmed only the increase in amygdala volume. A meta-analysis of gray matter alterations in bipolar disorder by Wise et al. (26) revealed significantly smaller GM volumes in patients relative to controls in the bilateral insula, superior temporal gyrus, medial prefrontal gyrus, and the anterior cingulate. Enlarged volumes were found in cerebellar regions, the bilateral middle frontal gyrus, the right middle and inferior temporal gyrus, and the right middle occipital gyrus.

In summary, there is ample evidence for structural abnormalities in schizophrenia, with the most robust findings showing an enlargement of the lateral ventricles (LV) and a volume reduction of the left superior temporal gyrus and the frontal brain, mainly in the prefrontal and orbitofrontal regions (27). However, a GM increase in the striatum and GM reductions in several other key structures have also repeatedly been reported. Literature on affective psychoses supports the existence of structural differences with GM increase (e.g., concerning the amygdala) as well as decrease (including the medial prefrontal and insular cortex) compared to controls.

#### Effects of antipsychotic medication

Antipsychotics may affect progressive brain changes during the course of the illness (28, 29). Fusar-Poli et al. (30) observed that at baseline, patients showed smaller whole brain volumes and larger LV than controls. There were progressive GM volume reductions and LV enlargements in patients but not in controls, even when controlling for illness-related factors. Antipsychotic medication has also been shown to increase striatal GM, explaining the finding of increased striatal GM in individuals with schizophrenia (21). In their meta-analysis, Goodkind et al. (21) reported no association between antipsychotic medication and insular volume. However, despite this emerging literature, the relationship between structural alterations, illness-, and treatment-related factors has not yet been sufficiently disentangled.

### Structural MRI findings in aggression

#### Structural MRI correlates of aggression in healthy persons

Matthies et al. (31) observed a negative correlation between amygdala volumes and aggression scores in healthy volunteers.

Sakuta and Fukushima (32) and Bufkin et al. (33) found the prefrontal cortex and medial temporal regions to be associated with aggression and explained their findings in the context of negative emotion regulation.

#### Structural MRI correlates of aggression in patient populations

Most of the research on structural correlates of aggression is based on patients with antisocial personality disorder or psychopathy.

Yang and Raine (34), in a meta-analysis, found reduced right orbitofrontal cortex (OFC), right anterior cingulate cortex, and left dorsolateral prefrontal cortex volumes in antisocial individuals.

In a review, Weber et al. (35) reported volume loss in prefrontal and right superior temporal gyrus, in the amygdala, and in the posterior hippocampus, as well as an increase in callosal white matter volume, in psychopaths. Psychopathy may be associated with brain abnormalities in a prefronto-temporo-limbic circuit—regions involved in emotional and learning processes (35).

Wahlund et al. (36) reported in a review that some studies showed smaller volumes in temporal regions, and some in frontal regions, while others found no differences. Raine et al. (37) observed increased corpus callosum volume in highly psychopathic antisocial subjects compared to that of healthy controls.

Aoki et al. (38) conducted a voxel-based meta-analysis on structural correlates in persons with antisocial behavior. They reported significantly smaller GM volumes in the left superior frontal gyrus, the left anterior insula, and the right lentiform nucleus in individuals with antisocial behavior, as compared to healthy controls. Larger volumes were reported in the right fusiform gyrus, the right inferior parietal lobule, the left superior parietal lobule, the right cingulate gyrus, and the right postcentral gyrus. In summary, findings regarding structural correlates of aggression in patients suggest a reduction in prefrontal and temporal volume as compared to healthy controls.

### Preliminary work on structural MRI correlates of aggression in patients with psychoses

Relatively few studies have examined structural MRI correlates of aggression in psychoses. Reviews have attempted to compile the available data: Naudts and Hodgins (39) observed that violent as opposed to non-violent persons with schizophrenia have impaired orbitofrontal functioning and hypothesize that structural brain abnormalities may exist in the amygdala-orbitofrontal system and in the prefrontal cortex and hippocampus. Soyka (27) and Hoptman et al. (40) state that frontal and temporal abnormalities are a consistent feature of aggression in schizophrenia.

In summary, published reviews suggest that there are structural alterations in aggressive, psychotic individuals. However, literature regarding affective psychoses is sparse. Existing reviews were conducted 5 or more years ago and are narrative expert reviews that lack systematic literature searches as well as reports of effect sizes. No systematic review (i.e., according to the PRISMA guidelines) or effect size analysis has been conducted on the topic to date.

### Objective and research question

Considering the high clinical importance of aggression in psychoses and the state of the literature, our aim was to conduct a systematic literature search, to compile all available data on structural MRI correlates of aggression in patients with psychotic disorders, and to calculate effect sizes of the observed differences. More specifically, we aimed at identifying structural magnetic resonance imaging differences in brain volumes between violent vs. non-violent persons with psychoses.

## Methods

To achieve a high standard of reporting we adopted the “Preferred Reporting Items for Systematic Reviews and Meta-Analyses” (PRISMA) guidelines (41) and the revised “Quality of Reporting of Meta-analyses” QUORUM statements (42). We registered the detailed study protocol on the International Prospective Register of Systematic Reviews database (PROSPERO; registration number: CRD42014014461) prior to the completion of data extraction (43). We assessed methodological quality using the PRISMA 2009 checklist (41). Out of 27 items, 23 were fulfilled, indicating an overall high methodological quality.

### Quality assessment

Quality of the studies was assessed using an item-checklist constructed specifically for the current work, similar to the quality assessment described by Paulson and Bazemore (44) and adapted by Fusar-Poli et al. (30). We rated precision, directness, and consistency of the data. The quality assessment categories are listed in Supplementary Table 1 (0–2 points per item, with a theoretical range of 0–38 for the total quality score). The included studies were characterized as high-quality (above 80% of the maximal sum of points), moderate-high (60–79%), moderate (40–59%), moderate-low (20–39%), and low-quality studies (below 19%). Nine of the 12 studies included in the meta-analysis had a moderate-high quality and three had a moderate quality.

Established and adapted quality checklists tend to reference the current state of the literature to at least some degree, and quality assessments of the included studies were rated as low to high quality with respect to the currently published manuscripts. For example, none of the published studies in the field has used longitudinal study designs, and controlling for lifetime substance use disorder is a difficult challenge not yet adequately met. Both factors would have further improved the quality of the included studies, but they are not currently accounted for in the quality assessment checklists.

### Search and selection strategy

We first conducted a systematic review of structural MRI studies on correlates of aggression in psychotics vs. healthy controls. We searched the PubMed, EMBASE, ScienceDirect, and PsycINFO databases, with no restriction on the publication start date range, and searched for publications through September 2017. We used search thesauri representing aggression, psychosis and brain imaging. The detailed search terms are available at http://www.crd.york.ac.uk/PROSPERO/display_record.asp?ID=CRD42014014461. Furthermore, we searched the reference lists of all of the selected original articles for additional literature.

We screened all studies according to the following inclusion criteria. We included longitudinal, cross-sectional, and case-control studies (journal articles, book chapters, and dissertations) reporting brain imaging correlates of aggression, comparing: (1) affective or non-affective psychosis groups with a history of violence, or including continuous measures of aggression, (2) affective or non-affective psychosis groups with a history of violence or including continuous measures of aggression, compared to healthy controls, (3) affective or non-affective psychosis groups with a history of violence or including continuous measures of aggression, compared to controls with diagnoses other than affective or non-affective psychoses, (4) affective or non-affective psychosis groups with a history of violence compared to affective or non-affective psychosis groups without a history of violence. Furthermore, we included all brain imaging studies using structural MRI with an age of cases and controls of at least 18 years. We applied no language restriction and required patients to have an established diagnosis of affective or non-affective psychosis according to DSM or ICD.

If there was insufficient information to extract the necessary data, we excluded the study. For the quantitative analyses, we excluded studies with no comparison group or with an overlap of more than 10% with the cases or controls reported in other studies selected, in which case, we then excluded the study with the smaller sample size. The entire process was conducted independently by two reviewers (SW, HAJ). In case of disagreement, reviewers discussed their reasons for inclusion or exclusion. If consensus was not reached, a third reviewer (CGH) was included, in order to reach a decision.

### Data extraction

The main outcome measure was whole brain volume of the two patient groups [violent persons with schizophrenia (VS) and non-violent persons with schizophrenia (NVS)] and healthy controls (HC), while the additional outcome measures were the specific regional volumes of the mentioned groups, reported in *mean* and *standard deviation (SD)*.

We extracted the following information from all studies: imaging center, first author, year of publication, type of imaging analysis, population characteristics of the healthy controls and patient groups (group size, gender, age, psychopathology, IQ, medication), and diagnosis. For an overview, see Table 1. All corresponding authors of the included publications were contacted by email using a standardized questionnaire for completing our coding sheets collecting means and standard deviations of whole brain volumes and specific regional volumes of all examined groups, as well as descriptive information about subjects.

**Table 1:**
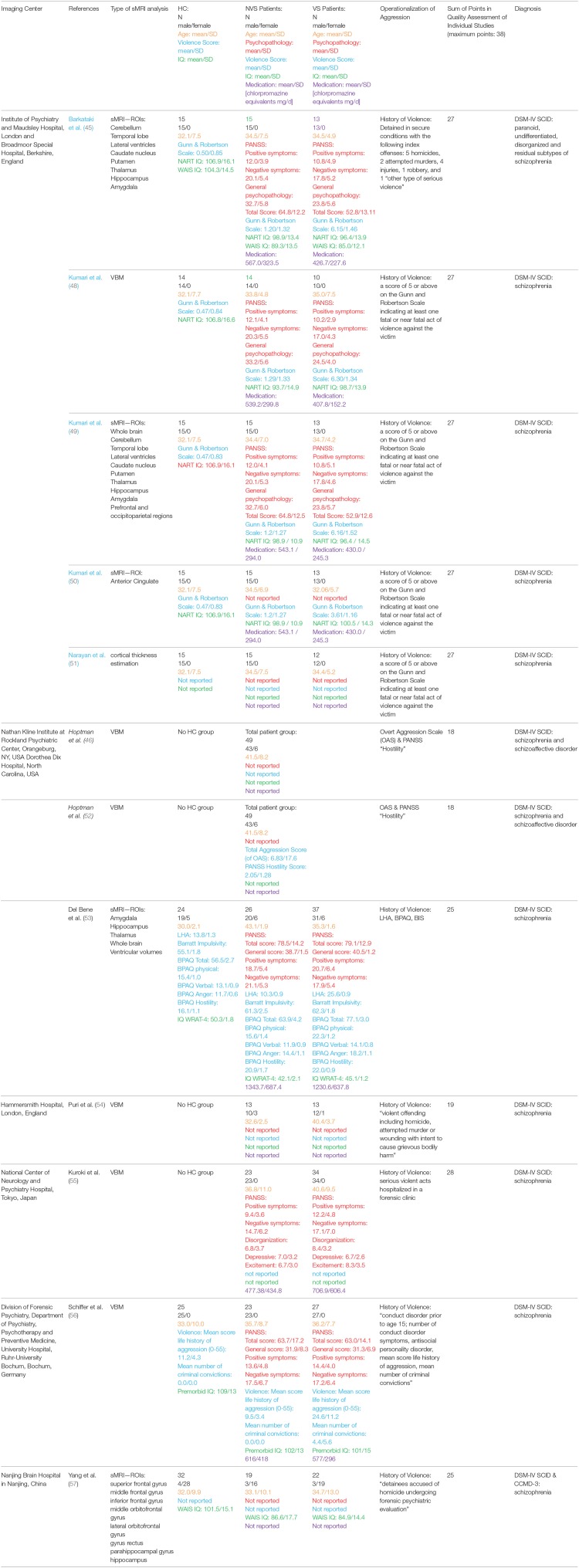
Overview of the studies included in the qualitative report, describing the imaging center; name of the first author; year of publication; type of imaging analysis; population characteristics of the healthy controls and patient groups (group size, gender, age, psychopathology, violence score IQ, medication); diagnosis.

*MRI, magnetic resonance imaging; sMRI, structural magnetic resonance imaging; VBM, voxel based morphometry; ROI, region of interest; HC, healthy controls; NVS, non-violent schizophrenia group; VS, violent schizophrenia group; HoV, History of Violence; PANSS, Positive and Negative Syndrome Scale; NART IQ, National Adult Reading Test for estimating premorbid intelligence levels; WAIS IQ, Wechsler Adult Intelligence Scale to measure cognitive ability in adults, Gunn & Robertson Scale, 5-item scale for rating violence and other criminal activities; Overt Aggression Scale OAS, 6-item scale for rating aggression (from verbal to physical); DSM-IV, Diagnostic and Statistical Manual of Mental Disorders; fourth edition; SCID, Structured Clinical Interview for DSM disorders; CCMD-3, Chinese Classification of Mental Disorders, third edition; LHA, Life History of Aggression; BPAQ, Buss-Perry Aggression Questionnaire; BIS, Barratt Impulsiveness Scale. Articles with author names marked in blue or in italic, respectively; used the same cohort, of which the work by Barkataki et al. (45) and Hoptman et al. (46), respectively, were the underlying primary studies*.

SW and HAJ independently searched the databases and extracted the relevant data in order to avoid bias or error in article selection and information coding.

### Data analysis

First, we performed a qualitative analysis of all included publications. Second, in a meta-analytic approach, we calculated (a) effect size separately for each study and (b) mean Hedge's g for global brain volume measurements and regions of interest including volumetric and morphometric results. All analyses were performed with “Statistical Package for Social Sciences” (IBM SPSS Statistics for Windows, version 23.0, IBM Corp., Armonk, NY, USA).

#### Group comparisons

We performed a one-way ANOVA to describe group characteristics with regard to sample size, gender, age, and IQ.

#### Meta-analysis

For computations, we used the SPSS macros written by Lipsey and Wilson (47). We calculated the pooled standard deviation, then standardized the mean effect size from statistical information reported in the studies. Due to small sample sizes (samples with less than 20 subjects) we corrected for this bias using Hedge's method, to receive an unbiased effect size estimate. We then calculated the effect size for each study separately using the unbiased effect size estimate. Finally, we weighted the effect size depending on each group's sample size.

## Results

### Literature search

**Figure 1:**
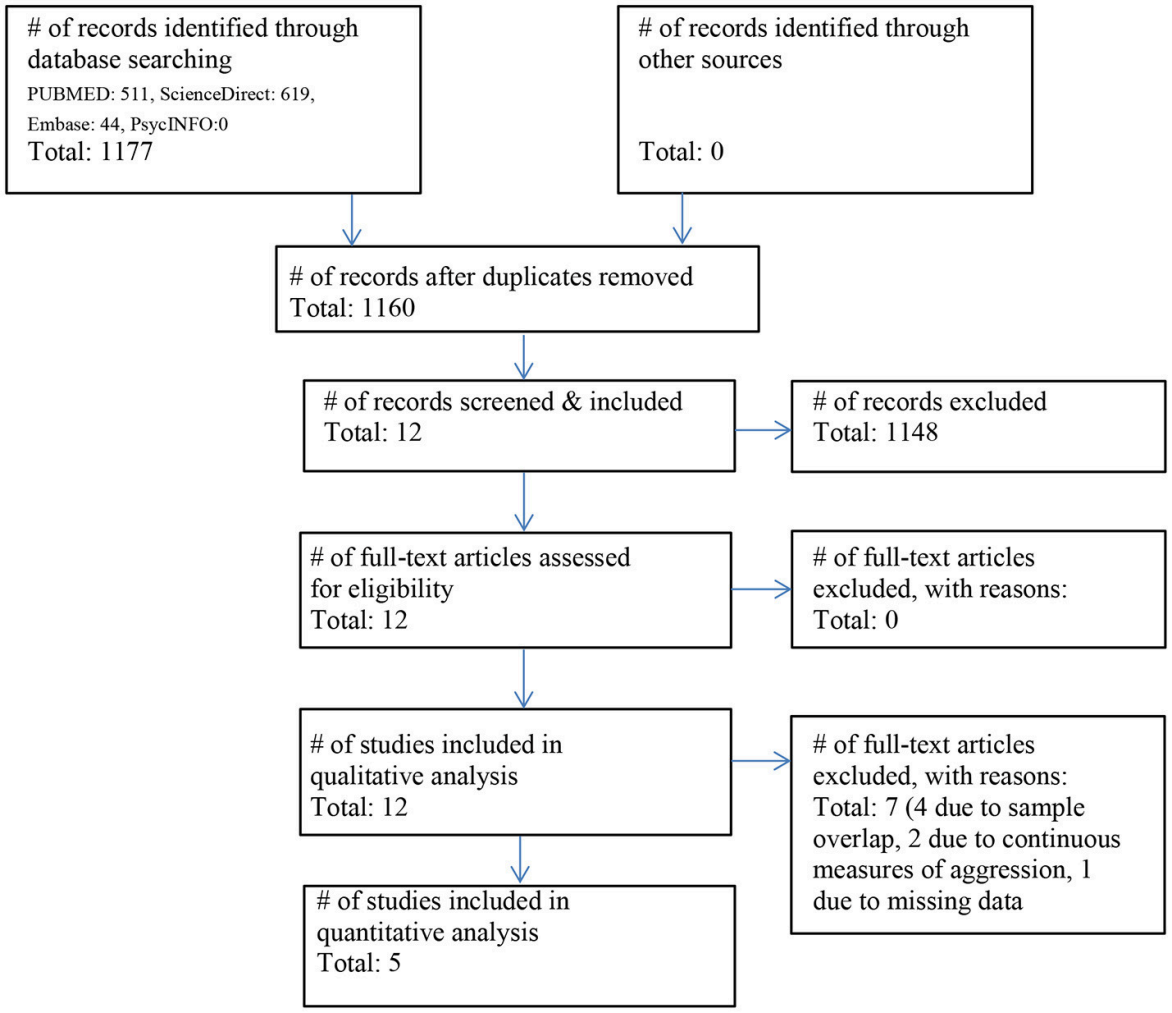
Flowchart of the literature search and included studies according to the PRISMA guidelines (41).

The initial literature search identified 1177 possible studies of interest. After screening all studies and applying inclusion and exclusion criteria, 1148 studies were excluded. Using the template of the PRISMA flow diagram, the study selection procedure is summarized in Figure 1. We found no studies including subjects with affective psychoses.

The final sample consisted of 12 studies with a total of 470 patients and 155 HC. After subtracting subject overlaps due to the publication of multiple papers using the same cohort, the sample consisted of 314 patients and 96 HC.

Table 1 gives an overview of all included studies showing the imaging center, name of the first author, year of publication, type of imaging analysis, population characteristics of HC, patient groups (group size, gender, age, psychopathology, IQ, medication), and diagnosis, also indicating sample overlap where applicable.

### Sample descriptives

Among the included 12 studies, there were no significant differences in sample sizes, age or gender across the groups (see Table 2). Regarding WAIS IQ we found significant differences between HC and NVS as well as VS. NVS and VS did not significantly differ in IQ (see Table 2). Missing data impeded calculation of differences regarding antipsychotic medication or psychopathology.

**Table 2:** Descriptive statistics of healthy control group (HC), non-violent schizophrenia patients (NVS), and violent schizophrenia patients (VS) over all 12 included studies, with the exclusion of overlapping cohorts.

	**HC *M (SD)***	**NVS *M (SD)***	**VS *M (SD)***	**Group differences (ANOVA) *p***
Sample size	24.0 (6.9)	20.4 (4.9)	24.4 (9.3)	0.568
Average % male	72.9 (41.4)	79.6 (30.0)	82.5 (31.1)	0.898
Age	31.7 (1.2)	36.7 (4.1)	37.6 (3.1)	0.033
IQ	102.9 (2.0)	88.0 (1.9)	84.9 (0.1)	0.003^+^

*HC, healthy control group; NVS, non-violent schizophrenia patients; VS, violent schizophrenia patients; M, mean; SD, standard deviation. Note. ^+^The group comparisons VS vs. HC and NVS vs. HC showed significant group differences in IQ [F_(2, 3)_ = 73.119] while the groups VS vs. NVS did not differ significantly in IQ*.

### Aggression operationalized as “history of violence”

Barkataki et al. (45), Kumari et al. (48–50), Narayan et al. (51), Del Bene et al. (53), Puri et al. (54), Kuroki et al. (55), Schiffer et al. (56), and Yang et al. (57) used the following three groups to examine aggression in schizophrenia: (1). Healthy, non-violent controls (HC), (2). Non-violent persons with schizophrenia (NVS), and (3). Violent persons with schizophrenia (VS).

**Figure 2:**
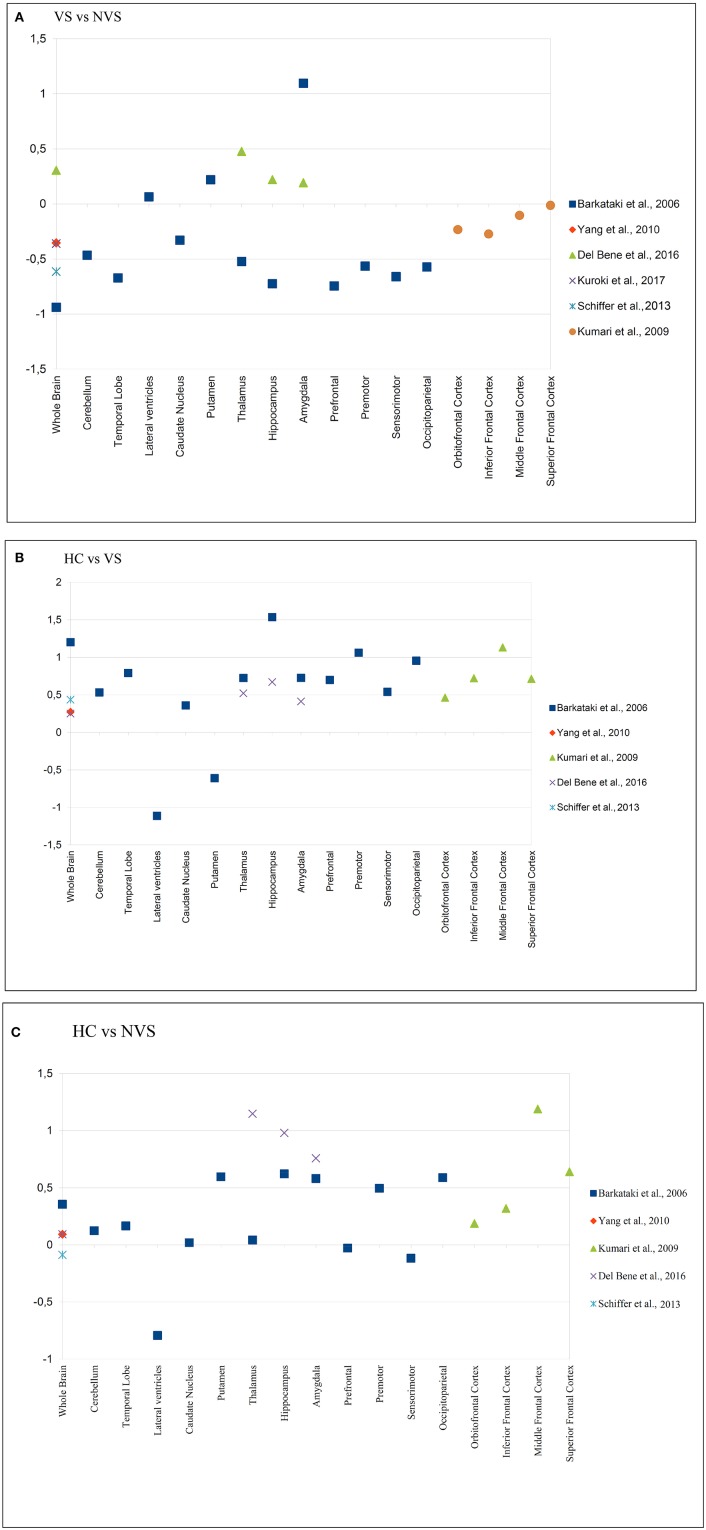
Effect sizes of reported brain areas in different studies. **(A)** Group comparison of HC vs. NVS; **(B)** Group comparison of HC vs. VS; **(C)** Group comparison of VS vs. NVS. A positive value indicates that the first group has a greater volume than the second group in the comparison e.g., in **(A,B)**, the HC group, and in **(C)**, the VS group. HC, healthy controls; NVS, non-violent schizophrenia group; VS, violent schizophrenia group. Barkataki et al. (45), Del Bene et al. (53), Yang et al. (57) have used ROIs, while Kumari et al. (48), Kuroki et al (55), and Schiffer et al. (56) applied VBM.

Kumari et al. (48–50), Narayan et al. (51) published work based on the cohort originally examined by Barkataki et al. (45). As part of the quantitative analyses, we calculated effect sizes for the comparison of HC vs. NVS, HC vs. VS, and NVS vs. VS where applicable. For an overview of effect sizes for each area, see Figure 2.

#### Violent vs. non-violent persons with schizophrenia

Most studies found decreased volumes in VS vs. NVS (45, 48, 49, 51, 54, 55, 57) while others found increased volumes in VS as opposed to NVS (49, 53, 56). For an overview, see Table 3.

**Table 3:** Overview of the qualitative findings comparing volumes in violent vs. non-violent schizophrenia patients OR (in *italic*) of continuous measures of aggression in schizophrenia patients.

**References**	**WB**	**Cer**	**TL**	**I**	**LV**	**CN**	**P**	**T**	**Hypo**	**Hip**	**Am**	**PFC**	**PMC**	**SMC**	**InfP**	**OPC**	**AC**	**OFC**	**InfF**	**MidF**	**SupF**	**PHG**
Barkataki et al. (45)	↓	↓	↓		↑	↓	↑	↓		↓	↑	↓	↓	↓		↓						
*Hoptman et al. (46)*																		↑				
*Hoptman et al. (52)*						↑																
Narayan et al. (51)														↓								
Kumari et al. (48)			↓							↓	↑							↓	↓	↓	↓	
Kumari et al. (49)	↓		↓							↓	↑											
Kumari et al. (50)																	↔					
Puri et al. (54)	↓	↓																				
Yang et al. (57)	↓									↓												↓
Del Bene et al. (53)	↑							↑		↑	↑											
Kuroki et al. (55)	↓		↓	↓																		
Schiffer et al. (56)	↓						↑		↑						↑				↓			

*WB, Whole Brain; Cer, Cerebellum; TL, Temporal Lobe; I, Insula; LV, Lateral Ventricles; CN, Caudate Nucleus; P, Putamen; T, Thalamus; Hypo, Hypothalamus; Hip, Hippocampus; Am, Amygdala; PFC, Prefrontal Cortex; PMC, Premotor Cortex; SMC, Sensorimotor Cortex; InfP, Inferior Parietal Cortex; OPC, Occipitoparietal Cortex; AC, Anterior Cingulate; OFC, Orbitofrontal Cortex; InfF, Inferior Frontal Cortex; MidF, Middle Frontal Cortex; SupF, Superior Frontal Cortex; PHG, Parahippocampal Gyrus. Articles marked blue used the same cohort, of which the work by Barkataki et al. (45) was the underlying primary study—The two studies by Hoptman et al. used the same cohort, as well (marked in italic). Red shading refers to relatively decreased volumes in violent schizophrenia vs. non-violent schizophrenia patients. Green shading refers to relatively increased volumes in violent schizophrenia vs. non-violent schizophrenia patients. Yellow shading refers to no significant differences in volumes between violent schizophrenia vs. non-violent schizophrenia patients*.

More specifically, Barkataki et al. (45) found that VS had a significantly reduced whole-brain volume compared to NVS. The VS group showed significantly larger putamen and smaller amygdala volumes than the NVS group—these findings, however, were not sustained when covarying for Positive and Negative Syndrome Scale (PANSS) general psychopathology score. In the study by Kumari et al. (48), temporal lobe and hippocampal volume were reduced in VS compared to NVS at a trend level. Kumari et al. (49) found that VS had smaller whole brain, temporal lobe, and hippocampus volumes than NVS. However, VS had larger amygdala volumes than NVS. When comparing the AC volumes of VS with NVS, there was no significant difference (50). Narayan et al. (51) found reduced cortical thickness in the right ventromedial prefrontal and lateral sensorimotor cortex in aggressive vs. non-aggressive people with schizophrenia.

Yang et al. (57) found reduced GM volume in the whole brain, hippocampus, and parahippocampal gyrus in VS compared with NVS. Puri et al. (54) reported that VS had smaller GM volume in the cerebellum than NVS and hypothesized that the cerebellum might be relevant for input from ventrolateral prefrontal cortex and parietal regions. Kuroki et al. (55) reported that VS as opposed to NVS showed significantly smaller volumes of the right inferior temporal area and the right insular area.

Schiffer et al. (56) observed that VS vs. NVS had increased volumes of the hypothalamus, left putamen, and right inferior parietal cortex. VS as opposed to NVS had smaller volumes of the inferior frontal region and also smaller whole brain volumes.

Del Bene et al. (53) reported larger whole brain volumes in VS as opposed to NVS. In the same group comparison, they found larger volumes in the amygdala, the hippocampus, and the thalamus.

In the group comparison of VS vs. NVS, we noted large negative effects on volume in the whole brain, temporal lobe, thalamus, hippocampus, prefrontal cortex, premotor cortex, sensorimotor cortex, and occipitoparietal cortex. In the same group comparison, we found large positive effects on volume in the amygdala (see Figure 2A).

#### Violent persons with schizophrenia vs. healthy controls

Kumari et al. (48) found significantly smaller PFC and hippocampal volume, and—at a trend level—smaller temporal lobe and amygdala volume in VS compared to HC. Kumari et al. (50) found significantly lower AC volumes in VS than in HC. When comparing HC vs. VS, we observed large positive effects on whole brain volume, cerebellum, temporal lobe, thalamus, hippocampus, amygdala, prefrontal cortex, premotor cortex, occipitoparietal cortex, and inferior, middle, and superior frontal cortex. In that group comparison, we found large negative effects in the LV and the putamen (see Figure 2B).

#### Non-violent persons with schizophrenia vs. healthy controls

NVS showed a trend for lower AC volumes than HC. The authors found no other significant group differences in volumes (50). Also, NVS showed smaller GM volume in prefrontal cortex than HC (57).

Kumari et al. (48) reported reduced PFC and amygdala volume in NVS compared to HC; furthermore, they observed that impulsiveness, as measured by the Impulsiveness-Venturesomeness-Empathy questionnaire [IVE-7, (58)], correlates negatively with reduced orbitofrontal GM volume. They hypothesized that dysfunctional, but not functional, impulsivity is elevated in repetitively violent persons with schizophrenia, and that this reduction in orbitofrontal GM may constitute a correlate of dysfunctional impulsivity.

When comparing HC vs. NVS we discovered large positive effects on volume in the putamen, hippocampus, amygdala, occipitoparietal cortex, middle frontal cortex, and superior frontal cortex. In the same group comparison, we found large negative effects in the LV (see Figure 2C).

### Aggression operationalized by means of questionnaires

Hoptman et al. (46, 52) used continuous measures to examine structural correlates of violence in schizophrenia in one subject population.

Hoptman et al. (46) found larger GM volumes in the left OFC to be associated with a higher degree of aggression as rated in the PANSS and Overt Aggression Scale (OAS). Also, larger GM volumes in the right OFC were associated with worse neuropsychological performance. The authors discussed the possibility that an increase in volume could constitute a correlate of reduced neural density, of increased neuronal size, of edema, or other pathophysiological processes. Hoptman et al. (52) reported that aggression in treatment-resistant schizophrenia or schizoaffective disorder is associated with a larger caudate volume.

In summary, studies measuring aggression by continuous means (using questionnaires) found increased volumes in the OFC as well as the caudate (46, 52) (see Table 3).

### Effect size analysis

After excluding all studies with overlapping cohorts, insufficient data, or missing comparison group, four studies suitable for meta-analysis of effect size remained (see Figure 1). We calculated an effect size analysis over whole brain volumes as reported in the studies by Barkataki et al. (45), Del Bene et al. (53), Kuroki et al. (55), Schiffer et al. (56), and Yang et al. (56) (see Table 4).

**Table 4:** Effect size analysis of whole brain volume in HC vs. NVS and HC vs. VS as measured in the studies by Barkataki et al. (45), Del Bene et al. (53), Schiffer et al. (56), and Yang et al. (57).

	**HC vs. NVS**				**HC vs. VS**				**NVS vs. VS**			
	***n***	***Mean Effect Size***	***P***	***Q***	***n***	***Mean Effect Size***	***p***	***Q***	***n***	***Mean Effect Size***	***p***	***Q***
			***p***				***p***	***q***			***p***	***q***
Whole Brain	4	0.0356	0.8140	1.1197	4	0.4223	0.0042	4.0581	5	0.3555	0.0073	5.2540

*In the group comparison NVS vs VS we could additionally include the study by Barkataki et al. (45), Del Bene et al. (53), Kuroki et al. (55), and Yang et al. (57) have used ROIs, while Kuroki et al. (55) and Schiffer et al. (56) applied VBM. n, number of studies; p, value of probability; Q, Homogeneity Coefficient*.

We observed that HC showed larger whole brain volumes than persons with schizophrenia, independently of their history of violence. In addition, studies revealed that VS had smaller whole brain volumes than NVS.

## Discussion

With this systematic review and effect size analysis, we sought to compile all available data on structural MRI correlates of aggression, comparing persons with schizophrenia and healthy controls. This is the first systematic qualitative and quantitative review on this topic. To ensure high methodological quality, it was conducted according to the PRISMA guidelines and the revised QUORUM statements.

### Qualitative results

#### Non-violent schizophrenia vs. violent schizophrenia

Volumes of whole brain, as well as cerebellum, temporal lobe, caudate nucleus, thalamus, hippocampus, prefrontal cortex, premotor cortex, sensorimotor cortex, occipitoparietal cortex, OFC, inferior, middle, and superior frontal cortex, and parahippocampal gyrus, are reported to be smaller in VS vs. NVS.

The parahippocampal gyrus volume reduction is specific to this group comparison. This structure is known to play an important role in scene recognition (59). This ability is reported to be impaired in violent persons (60) and also in persons with schizophrenia (61). Also, the parahippocampal gyrus seems to be involved in emotion processing (62). The authors, in a lesion study, detected that the structure may play a crucial role in comprehending social context (e.g., sarcasm). Poorer sarcasm comprehension was correlated with smaller parahippocampal gyrus volumes (62). An impaired comprehension of hidden meanings of communication in social contexts may lead to misunderstandings and, thus, may be connected with aggressive actions. Our finding is therefore compatible with the literature, while its specificity to aggression remains unclear.

The putamen, LV, and amygdala are reported to be larger in VS vs. NVS. The putamen is known to play a core role in regulating movement and learning abilities (63–65). Also, Zeki and Romaya (66) have reported the putamen to be hyperactivated when viewing a hated face vs. a neutral face. The increased putamen volumes in violent individuals may reflect an association with feeling hatred—still, antipsychotic pharmacotherapy is a potential moderator of this effect. In general, aggressive persons receive higher medication doses, and it is known that the intake of antipsychotic medication increases putamen volume (67, 68).

Amygdala volume has been reported to be reduced in aggressive or non-aggressive persons with schizophrenia as opposed to healthy controls. We find a larger amygdala volume in VS than in NVS. This finding is novel in the literature. The amygdala, as part of the limbic system, plays an important role in developing fear and in emotion regulation (69). A possible interpretation of our findings could be that the reductions in amygdala volumes are more pronounced in NVS than in VS, which could lead to the hypothesis that the amygdala is more prominent in aggression. Pardini et al. (70), in contrast, found lower amygdala volume to be associated with aggression.

There is no significant difference reported on anterior cingulate volume between VS and NVS.

Studies comparing persons with schizophrenia with higher or lower aggression scores in questionnaires found larger volumes in the caudate nucleus and in the OFC in persons with schizophrenia with higher aggression scores vs. those with lower aggression scores.

There are contradictory results concerning the OFC and caudate nucleus: Hoptman et al. (46) reported higher OFC volume while Kumari et al. (48) reported the OFC volume to be lower. Also, Hoptman et al. (52) found the caudate nucleus to be larger, while Barkataki et al. (45) reported this volume to be smaller. This difference may be attributable to the different study designs: both Hoptman papers examined aggression as a continuous measure while the reports by Barkataki and Kumari used categorical measures. Therefore, the contradictory findings could be attributed to the substantially different operationalization of aggression or potentially to medication effects.

Persons with schizophrenia independently of aggressive behavior show a general volume reduction in the whole brain and an increase in ventricular volumes. It remains unclear whether the differences in volumes described above are attributable to the aggressive behavior or to effects of disease or medication.

#### Healthy controls vs. violent schizophrenia persons

When comparing healthy controls with violent persons with schizophrenia, we found smaller volumes of the whole brain, cerebellum, temporal lobe, caudate nucleus, thalamus, hippocampus, amygdala, prefrontal cortex, premotor cortex, sensorimotor cortex, occipitoparietal cortex, OFC, and inferior, middle, and superior frontal cortices. In contrast, the putamen and LV were larger in violent persons with schizophrenia than in healthy controls.

Most of these differences equal those described for the comparison between healthy controls and non-violent persons with schizophrenia (17, 19, 20, 22, 39).

However, differences pertaining to the prefrontal cortex, sensorimotor cortex, and the putamen are specific to this group comparison.

We found the prefrontal cortex volume to be reduced in aggressive persons with schizophrenia. The prefrontal cortex is known to play an important role for the regulation of emotion (71, 72). We know emotional regulation to be impaired in aggressive persons (72). Our finding of reduced prefrontal cortex volume is therefore compatible with the current literature.

We found reduced sensorimotor cortex volume in violent persons with schizophrenia. This structure plays an important role in planning and executive motor functioning (73). We know that these abilities are impaired in persons with schizophrenia (74) and in aggression (75), and conclude that our finding of reduced sensorimotor cortex volume is also consistent with the literature.

We found increased putamen volume in violent persons with schizophrenia. As described above, the putamen volume increase plays an important role in feeling hatred, but might also be an effect of antipsychotic medication.

#### Healthy controls vs. non-violent schizophrenia persons

When comparing healthy controls with non-violent persons with schizophrenia we found smaller whole brain volume and smaller volumes of the cerebellum, temporal lobe, caudate nucleus, putamen, thalamus, hippocampus, amygdala, premotor cortex, occipitoparietal cortex, OFC, and the inferior, middle, and superior frontal cortices in the schizophrenia group. Non-violent persons with schizophrenia had larger LV than the healthy controls. These findings are in line with the existing literature where Wright et al. (19) observed that the mean cerebral volume of persons with schizophrenia was smaller than in healthy controls, and that the total ventricular volume in persons with schizophrenia was enlarged in comparison to healthy participants. Shenton et al. (20) confirmed these findings by noting that persons with schizophrenia showed smaller WB volumes and enlarged ventricles. Also, they found amygdala, hippocampus, parahippocampal gyrus and neocortical temporal lobe regions to be smaller in persons with schizophrenia than in healthy controls. Fusar-Poli et al. (22) reported consistent GM reductions in temporal, anterior cingulate, cerebellar, and insular regions. In a meta-analysis, van Erp et al. (18) found smaller hippocampus, amygdala, thalamus, accumbens, and intracranial volumes, but larger lateral ventricles, in persons with schizophrenia as compared to healthy controls.

In the two comparisons of brain volume in VS and NVS as compared to HC, we consider the IQ score as a potential confounding factor. As IQ was significantly lower in the two patient groups as opposed to HC, this could further influence the reduced brain volumes in the patient groups. In a large-scale meta-analysis, McDaniel (76) reported a clear positive correlation between brain volume and intelligence.

### Meta-analytic results

Violent persons with schizophrenia showed a significantly lower whole brain volume than healthy controls (*p* = 0.0042), and non-violent persons with schizophrenia had a significantly larger whole brain volume than violent persons with schizophrenia (*p* = 0.0073).

These findings could be influenced by factors related to aggression or further confounders (e.g., publication bias)—it could be possible, for example, that they are the effects of violent persons with schizophrenia receiving higher cumulative doses of antipsychotic or sedating medication, or of differences concerning co-morbid substance use disorder. Unfortunately, due to sample size restrictions and the failure of most studies to report potential confounding variables, this question cannot be further explored based on the available literature.

Non-violent persons with schizophrenia had a lower whole brain volume than healthy controls, although this difference is not significant. This finding is only partially in line with the literature: We would have expected a significantly smaller whole brain volume in both violent and non-violent persons with schizophrenia compared to healthy controls (19). However, non-significance in this case might be caused by the small sample size.

### Challenges and pitfalls

There are several major challenges when examining structural correlates of aggression in psychoses that limit the explanatory power of our systematic review and effect size analysis.

#### Operationalizing aggression

Aggression in schizophrenia-spectrum disorders is difficult to examine due to the heterogeneous clinical picture of schizophrenia and the extensive variety of aggressive behaviors. In most of the reviewed articles, history of violence is not specified, and the scale of the violent acts remain unclear. This makes it impossible to control for the different types of aggression. Furthermore, from a clinical viewpoint, there are different trajectories of aggressive behavior (e.g., a number of minor aggressive events, one severe aggressive event, or a history of repeating aggressive events) that may be connected with different developmental pathways (e.g., repeated aggression in acute psychosis vs. development of a conduct or an antisocial personality disorder) and different neurobiological mechanisms of violence. Thus, further studies should aim to use narrow operationalizations of violence and to include more homogeneous patient populations to enable examination of these different pathways.

#### Predictors and moderators of aggression

There are many predictors of violent behavior (most importantly substance use disorder and antisocial personality disorder), however, the included studies do not sufficiently report information on these predictors nor do they systematically control for them. Therefore, we could not control for important moderators like psychopathology, general intelligence, or effect of medication. We recommend that future studies collect and report predictors of aggression in order to allow for moderator analyses. The most important factors that should be recorded and reported are antipsychotic medication, substance abuse comorbidities, IQ, and antisocial personality disorder.

#### Sample size, cohort overlaps, methodology, and publication bias

In general, small sample sizes and considerable cohort overlaps limit the interpretability of the current literature. Concerning the effect size analysis, the limited number of studies leads to a reduced statistical power, and results have to be considered preliminary and subjected to replication analyses when the situation of the published data has improved. Furthermore, according to Sterne et al. (77), the minimum number of studies needed for creating a funnel plot in meta-analyses is 10. Thus, due to sparse literature in the field, the important question of publication bias cannot be appropriately addressed yet. In addition, the use of different methods in measuring brain volumes (ROI-based analyses vs. VBM) complicate the interpretation of the findings. Although Focke et al. (78) indicated that both methods deliver comparable results, these methodological differences constitute an issue that has to be handled with caution. Replication studies will be needed in order to enable a better interpretation of the findings.

#### Affective psychoses

With respect to our systematic literature search, there is no published literature on structural magnetic resonance correlates of aggression in affective psychoses. This is surprising since persons with affective psychotic disorders are also at increased risk for aggressive behavior (4–6).

## Conclusion

Violence in patients with psychosis is of high clinical and public relevance. We reviewed evidence for differences in brain volume correlates of aggression in persons with schizophrenia. However, due to considerable sample overlap in the literature and missing research on affective psychoses, further studies evaluating structural correlates of aggression in psychotic disorders are urgently needed. In order to enhance comparability of studies, we recommend that researchers adhere to clear and exact definitions of history of violence and report consistent measurements of psychopathology, including violence scores.

## Author contributions

CH, SB, and RS designed the study. SW and HJ independently conducted literature search and data extraction, and CH supervised this process and helped reach a decision in case of disagreement. SW and JS analyzed the data. SW and CH wrote the initial draft of the paper. All authors contributed to data interpretation and manuscript preparation, and all authors read and approved the final version of the manuscript. SW and JS had full access to all the data in the study and take responsibility for the integrity of the data and the accuracy of the data analysis.

### Conflict of interest statement

The authors declare that the research was conducted in the absence of any commercial or financial relationships that could be construed as a potential conflict of interest.
