# Supplementary material for: Structural Magnetic Resonance Imaging Correlates of Aggression in Psychosis: A Systematic Review and Effect Size Analysis
Source: Front Psychiatry. 2018 Jun 7;9:217. doi: 10.3389/fpsyt.2018.00217 (PMC6000417; doi:10.3389/fpsyt.2018.00217)
Supplement: Supplementary file 1 [file Table_1.DOCX]

| **Category** | **0 P** | **1 P** | **2 P** |
| --- | --- | --- | --- |
| Participant selection assessment and validation (ICD or DSM) | Not clearly stated | No | Yes |
| Controls have been recruited from same population as cases | Not clearly stated | No (e.g. selected population such as hospital staff or students) | Yes |
| Assessor is blind for disease status | No / Not clearly stated | - | Yes |
| Methods for assessment of aggression clearly stated | No | - | Yes |
| Inclusion criteria | Not reported | Partly reported | Reported |
| Exclusion criteria | Not reported | Partly reported | Reported |
| Gender | Not reported | - | Reported |
| IQ / educational level | Not reported | - | Reported |
| Race / ethnic origin | Not reported | - | Reported |
| Somatic comorbidities | Not reported | - | Reported |
| Substance use disorder & antisocial personality disorder as comorbidities assessed | Not reported | 1 reported | Both reported |
| Other psychiatric comorbidities | Not reported | - | Reported |
| Are analyses adjusted for potential confounders? | No | Partly | Yes |
| Duration of illness /age of onset | Not reported | - | Reported |
| Previous antipsychotic medication (PAM) | Not defined | - | Defined |
| Duration of PAM | Not defined | - | Defined |
| Psychopathology reported | Not reported | - | Reported |
| Statistical Threshold reported | Not reported | - | Reported |
| WB: MNI/Tal coor  ROI: interrater reliability | Not reported | - | Reported |

Table 1 Supplement

**Table 1 of the Supplement.** Risk of Bias Assessment according to the quality checklist of Fusar-Poli et al., 2013 (adapted).
